# Impact of dietary resistant starch type 4 on human gut microbiota and immunometabolic functions

**DOI:** 10.1038/srep28797

**Published:** 2016-06-30

**Authors:** Bijaya Upadhyaya, Lacey McCormack, Ali Reza Fardin-Kia, Robert Juenemann, Sailendra Nichenametla, Jeffrey Clapper, Bonny Specker, Moul Dey

**Affiliations:** 1 Health and Nutritional Sciences, South Dakota State University, Box 2203, Brookings, SD 57007, USA; 2 Office of Regulatory Science, Center for Food Safety and Applied Nutrition, HFS-717, US Food and Drug Administration, College Park, MD 20740, USA; 3 Department of Animal Science, South Dakota State University, Box 2170, Brookings, SD 57007, USA; 4 Ethel Austin Martin Program in Human Nutrition, South Dakota State University, Box 506, Brookings, SD 57007, USA

## Abstract

Dietary modulation of the gut microbiota impacts human health. Here we investigated the hitherto unknown effects of resistant starch type 4 (RS4) enriched diet on gut microbiota composition and short-chain fatty acid (SCFA) concentrations in parallel with host immunometabolic functions in twenty individuals with signs of metabolic syndrome (MetS). Cholesterols, fasting glucose, glycosylated haemoglobin, and proinflammatory markers in the blood as well as waist circumference and % body fat were lower post intervention in the RS4 group compared with the control group. 16S-rRNA gene sequencing revealed a differential abundance of 71 bacterial operational taxonomic units, including the enrichment of three *Bacteroides* species and one each of *Parabacteroides*, *Oscillospira*, *Blautia*, *Ruminococcus, Eubacterium*, and *Christensenella* species in the RS4 group. Gas chromatography–mass spectrometry revealed higher faecal SCFAs, including butyrate, propionate, valerate, isovalerate, and hexanoate after RS4-intake. Bivariate analyses showed RS4-specific associations of the gut microbiota with the host metabolic functions and SCFA levels. Here we show that dietary RS4 induced changes in the gut microbiota are linked to its biological activity in individuals with signs of MetS. These findings have potential implications for dietary guidelines in metabolic health management.

Metabolic syndrome (MetS) encompasses co-morbidities like obesity, dyslipidaemia, hypertension, insulin resistance, and hyperglycaemia, which increase the risk of cardiovascular diseases^1^, the leading cause of death in the US^2^. Although it is possible to manage these co-morbidities with dietary/lifestyle changes and/or therapeutic interventions, the overall prevalence of MetS is rising worldwide^2^,^3^. One contributing factor could be non-adherence to healthy dietary practices beyond the short term, as convenience and taste remain the strongest determinants of food choices^4^. In that context, our recent double-blind study allowed participants to maintain their habitual dietary practices during the intervention without adapting to any change^6^. This was possible due to the neutral and adaptable organoleptic properties of the resistant starch (RS) type 4 (RS4), a wheat-derived food-ingredient, used for the intervention.

Different chemical properties contribute to the functional differences between five RS types, particularly in terms of fermentability and its influence on the microbiota in the gut^7^,^8^. Fermentable carbohydrates such as RS4, may potentially increase colonic short-chain fatty acid (SCFA) production^9^. However, there remains a dearth of well-controlled holistic intervention studies that have comprehensively examined the influence of RS4 on host physiological, gut microbiome, and SCFA changes^10^,^11^,^12^. One study reported influence of RS4 in healthy individuals, unrelated to any metabolic condition^8^. Other published studies regarding metabolic health benefits of RS4 involved animal models^13^,^14^,^15^. However, due to various metabolic adaptations in MetS patients, they may not always benefit from the information generated in healthy humans or animal models^16^. Taken together, there is a critical need for well-designed studies in individuals with MetS that systematically connects the influence of a functional and adaptable food ingredient on the gut microbial community, bacterial metabolites, and host metabolic functions.

In our trial, the RS4-group had improved lipid profiles and body composition^6^. Since RS4 is indigestible, we hypothesized that the health benefits of RS4 are derived from its ability to influence the gut microbial community structure, which may, in turn, be linked to altered bacterial fermentation and SCFA production^17^. Therefore, here, in twenty selected participants with MetS, we retrospectively examined the changes in the microbiota composition and the SCFA production in the gut, measured the concentrations of three circulatory adipocytokine markers, and estimated the macronutrient and caloric intake during the intervention period. Also, the host anthropometric and metabolic parameters were reanalysed in this cohort to show microbe-microbe and host-microbe interactions.

## Materials and Methods

Detailed information on methods is provided as supplementary information.

### General study considerations and diet analysis

**Figure 1:**
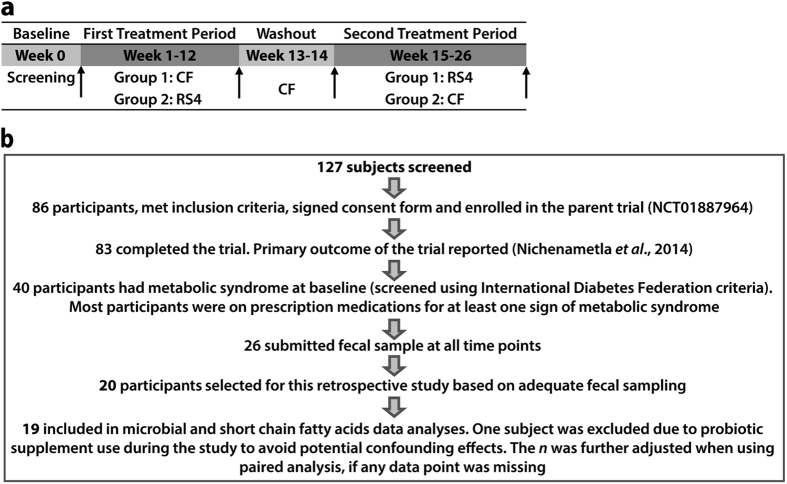
Study design. **(a)** Time line of the placebo-controlled, crossover, dietary intervention with resistant starch (RS4) and control flour (CF). Stool and blood samples were collected before and after each treatment period (indicated by arrows). **(b)** Trial profile and numbers of participants in the study.

This investigation involved 20 selected participants (Fig. 1) from the parent placebo-controlled, double-blinded, crossover, dietary intervention study^6^ (registered at clinicaltrials.gov as NCT01887964). The study duration was 26 weeks that included two 12-week interventions periods, with one each for RS4 (30%, v/v in flour) and control flour (CF), and a two-week washout in between the interventions. The participants from two North American Hutterite (Caucasian) communities were screened for MetS using the International Diabetes Federation criteria^18^. All procedures were conducted with the approval (1112012-CR) of the Institutional Review Board for Human Subjects Research of South Dakota State University and are in accordance with the Declaration of Helsinki. Informed consent was obtained from all participants before enrolment into the study. Diet information was collected using a self-administered semi-quantitative food frequency questionnaire and was analysed using Nutritionist Pro (Axxya Systems, Redmond, WA, USA). Anthropometric, blood lipids, glycaemic parameters, and blood pressure measurement data were obtained from the parent study and reanalysed in this sub-cohort^6^.

### Measurement of adipocytokine markers in blood

Serum interleukin-6 (IL6) and tumour necrosis factor-alpha (TNFα) were determined in pico-gram/mL using Human ELISA Ready-SET-Go kits (eBioscience, San Diego, CA), and plasma adiponectin concentrations were measured in micro-gram/mL using Human Adiponectin Radioimmunoassay (Linco Research, St. Charles, MO, following the manufacturer’s instructions.

### Gut microbial community structure analysis

Stool DNA was extracted using the QIAamp DNA Stool Mini Kit (QIAGEN, Valencia, CA) and outsourced to Second Genome (South San Francisco, CA) for 16S rRNA (V4 hypervariable region) gene sequencing using MiSeq instrument (Illumina, San Diego, CA). Taxa were filtered to those present in at least one of the samples to calculate relative abundance of a taxon within each sample. Differential abundance between the samples was calculated based on per million sequences in that sample. For unidentified Greengenes operational taxonomic units (OTUs), closest hits from NCBI 16S rRNA database were cross referenced with >90% query cover, >87% identity, and <0.001 E-value. Raw sequences are currently being deposited in NCBI sequence read archive (SRA, accession number SRP035338), belonging to BioProject accession number PRJNA308315.

### Faecal SCFA analysis by gas chromatography-mass spectrometry (GC-MS)

SCFAs were derivatized to their corresponding butyl esters (SCFABE) followed by GC-MS analyses using a GC-MS 5977A and HP-5MS UI capillary column from the same manufacturer (Agilent, Wilmington, DE, USA). The data were expressed in mg/gm of faecal sample.

### Statistical analyses

Data were analysed comparing end-points for outcome variables in CF and RS4 groups, or pre- and post-intervention measures. Linear mixed effects models (SAS MIXED procedure) were used to compare the effects of RS4 and CF on physiologic parameters as in our previous report^6^. All models included variables for colony and season, where colony was a surrogate for randomization sequence and season was a surrogate for crossover treatment period. General linear mixed models were also used to compare the effects of RS4 and CF on microbial abundance using R software package^19^. To correct for multiple comparisons, a false discovery rate (FDR or Benjamini Hochberg method) correction was used to adjust *p* values (adjusted *p* is represented as *q*). For pre- and post-intervention comparisons paired *t*-test (Wilcoxon signed-rank test for non-normal data) was used, while student’s *t*-test (Mann-Whitney signed-rank test for non-normal data) was used to compare two different diet groups. Where necessary, data were logarithmically transformed to achieve normality. Intra-relationships among parameters or bacterial species, and inter-relationships between parameters and microbiota were carried out using Pearson’s linear correlation coefficient (r). Correlation matrices and heat maps were generated using various R-packages. The data were presented as means ± S.E.M, unless otherwise noted. A *p* value of 0.05 or less was considered significant, while *p* value of 0.05 to 0.09 was considered trend or approaching significance.

## Results

### Baseline physiologic, metabolic, and microbiome characteristics of the study participants

All twenty participants who had signs of metabolic syndrome at baseline and submitted adequate stool samples at four data collection time points were included in the current investigation (Fig. 1), which allowed for comparison of the gut microbial and SCFA profiles before and after the interventions and also between the endpoints of the RS4 and CF (control) interventions. Potential adverse gastrointestinal side effects from the interventions were not evaluated in this cohort since none were observed in the parent cohort^6^. Baseline characteristics of 20 participants are summarized in supplementary Table S1. Taxonomic classification of a total of 55,079 sequences (present in at least one of the samples) were sorted into 5,949 OTUs, of which ~78% were associated with the phylum Firmicutes and ~9% with the phylum Bacteroidetes (Supplementary Fig. S1).

### Washout was effective in restoring microbiome characteristics

Before switching the RS4 and CF diets in the cross-over study design, all the participants were supplied with CF during the 2-week washout period in order to avoid the potential carry-over effects of the RS4 intervention. For endpoint comparison between the RS4 and CF groups, it was necessary to check for a consistent baseline prior to each treatment period. Using permutational multivariate analysis of variance for distance matrices, no significant differences were observed among the starting microbiomes of the RS4 and CF groups (data not shown), which also confirmed that the two-week washout was effective and that any differences observed post-intervention are due to the intervention itself.

### Macronutrient intake pattern did not vary during the study

Variation in macronutrient intake and total calories consumed can potentially influence the gut microbiota^20^, thereby confounding the effects of the intervention. Although a large number of food options are offered at each meal, Hutterites have relatively small interpersonal differences in diet due to common meal planning, kitchen, and dining practices. No significant differences in overall macronutrients and caloric intake were observed between the baseline and post-intervention time periods, with the exception of dietary fibre (Table 1). Dietary fibre intake, analysed separately from total carbohydrate intake, was significantly higher in the RS4 group (*p* < 0.001), due to RS4 being classified as a prebiotic dietary fibre (Table 1). The average calories (~1,774 Kilocalories) consumed at baseline were estimated to come from carbohydrate (~49%), protein (~17%), and fat (~34%). These values fall within the Dietary Reference Intakes (DRI) for macronutrients, which are 45–65%, 10–35%, and 20–35% for carbohydrate, protein, and fat, respectively^21^. Of particular interest, saturated fat (12.6%, DRI < 10%) and cholesterol (415 mg, DRI < 300 mg) intakes were higher, while daily fibre intake was lower (18 g at baseline, DRI 20–30 g) than recommended in the participants studied.

### Differential post-intervention effects of the RS4 diet compared with the CF diet on the gut microbiota

The current understanding is that, in studies without a proper control group, inter-individual variation in gut microbial composition in adults frequently offsets the smaller changes induced by dietary interventions^10^. To address this problem, we compared microbial composition and abundance post RS4 compared with post CF intervention. Three taxa, all unclassified species of Firmicutes, differentially shifted between CF and RS4 treatments in eight male participants. Similarly, a differential effect of RS4 was observed in 16 Firmicutes taxa, with the most numerous genus being *Enterococcus*, which was significantly enriched after CF intake in 12 female participants (data not shown). No distinct trend for Firmicutes to Bacteroidetes ratio was observed in male or female participants (data not shown). The dominance of Firmicutes and Bacteroidetes was consistent with previous results, as reported in Hutterite^22^ and other populations^23^. The Shannon diversity index was not associated with the age of the participants (r = −0.2, *p* > 0.05, data not shown). Likewise, the total diversity of the microbiota assessed from the Shannon diversity index did not significantly change after either CF or RS4 interventions (data not shown).

**Figure 2:**
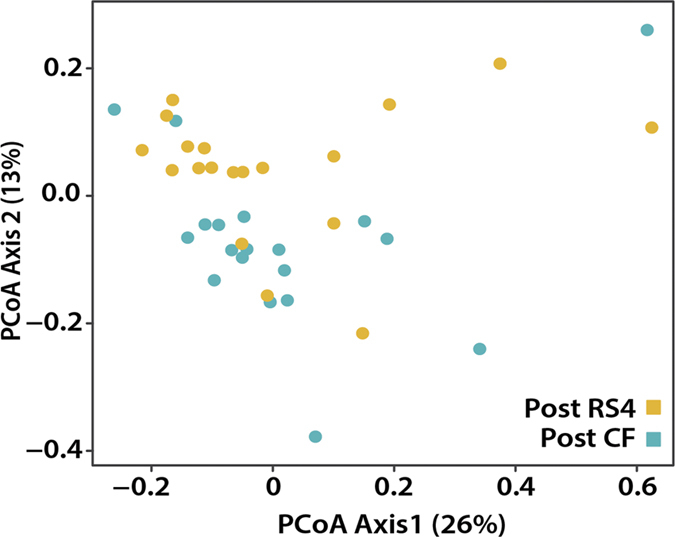
Separation of the microbiome post intervention in RS4 and CF groups. Two-dimensional principal coordinate analyses (PCoA) based on the weighted UniFrac distance between samples, given the abundance of 5,831 taxa present in at least one sample (n = 19). Axes 1 and 2 explain 26% and 13% of the variation, respectively (*p* = 0.01).

**Figure 3:**
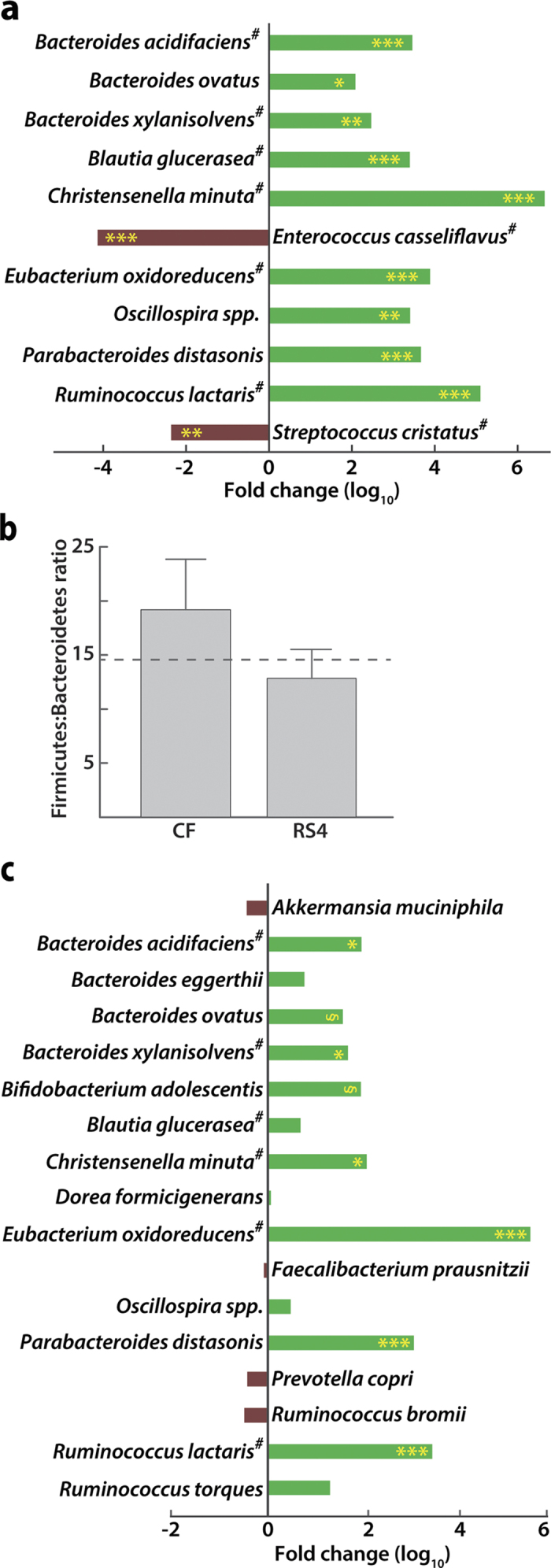
Differential gut microbial composition after RS4 intervention at the species level. **(a)** Relative abundance of bacterial species (log fold change) in the RS4 group compared with the CF group post intervention (n = 19). Significant compositional variation between the two groups before the intervention was previously ruled out. **(b)** The Firmicutes/Bacteroidetes ratio after intervention (n = 14). The dotted line represents this ratio at baseline. **(c)** Abundance of major bacterial species (log fold change) before and after RS4 treatment. #, the closest hit from the NCBI 16S rRNA database cross referenced with the OTU from the Greengenes database. *q ≤ 0.05, **q ≤ 0.01, ***q ≤ 0.001, ^§^*q* ≤ 0.09 (trend/approaching significance), n = 19.

Principal coordinate analysis showed 26% and 13% variations on axes 1 and 2, respectively, indicating a major shift between the two groups (*p* = 0.01, Fig. 2). The RS4 diet differentially modified 71 microbial OTUs (*q* < 0.05), including enrichment of four each of *Ruminococcus* and *Blautia*, two each of *Bacteroides* and *Oscillospira*, and one *Parabacteroides* OTUs (Supplementary Table S2). Of the 71 OTUs, 65 belonged to the phylum Firmicutes. The three Bacteroidetes OTUs all increased in abundance with RS4 relative to the CF treatment, while OTUs belonging to Firmicutes had a mixed response (Supplementary Table S2). At the species level, some species were significantly enriched in the RS4 group, including three *Bacteroides* species (>121.2 fold, *q* < 0.05) along with *Blautia glucerasea* (2497.1 fold, *q* < 0.001), *Christensenella minuta* (2.4 × 10^6^ fold, *q* < 0.001), *Eubacterium oxidoreducens* (7723.2 fold, *q* < 0.01), *Oscillospira* spp. (2528.4 fold, *q* < 0.01), *Ruminococcus lactaris* (1.2 × 10^5^ fold, *q* < 0.001), and *Parabacteroides distasonis* (8642.2 fold, *q* < 0.001), while some were significantly decreased in abundance in this group, including pathogenic *Enterococcus casseliflavus* (−13603.2 fold, *q* < 0.001) and *Streptococcus cristatus* (−229.7 fold, *q* < 0.05) (Fig. 3a). Although the enrichment fold changes for some of the bacterial species appear very high, their relative abundance in the whole microbial community could be low. This is due to the commonly used sampling normalization approach based on per million sequences to remove any bias due to varying sequencing depth (details in Methods). Overall, trends showed that Bacteroidetes OTUs were increased in the RS4 group, leading to an overall lowering of the average Firmicutes-to-Bacteroidetes (F:B) ratio in the RS4 group from 14.6 at baseline to 12.9, but increasing to 19.2 post CF diet (Fig. 3b). The lower F:B ratio is frequently perceived as an indicator of a leaner phenotype, although the previously reported results are not always consistent^24^. Firmicutes and Bacteroidetes are two major phyla and the species composition within each may vary widely in a given subject. It is possible that both phyla include species that may be characteristic of a particular phenotype. Therefore, a species level composition may represent a body weight phenotype more precisely than a broad estimation of F:B ratio.

### Impact of RS4 on gut microbiota composition compared before and after RS4 intervention

Firmicutes species from Clostridium cluster XIVa account for almost 60% of the mucin-adhered microbiota^25^. A general observation was that species from Clostridial cluster XIVa, but not cluster IV, were enriched by RS4 supplementation of the diet. At the species level (Fig. 3c), RS4 consumption increased the abundance of *Bifidobacterium adolescentis* (90.5 fold, *q* = 0.087) and *Parabacteroides distasonis* (1180.2 fold, *q* < 0.001) but not *Ruminococcus bromii* (−3.2 fold, *q* > 0.05), *Faecalibacterium prausnutzii* (−1.2 fold, *q* > 0.05), or *Dorea formicigenerans* (1.1 fold, *q* > 0.05), which confirmed the previous report^8^. Novel observations include an RS4-induced increase in *Christensenella minuta* abundance (119.7 fold, *q* = 0.038, 97% query coverage, 88% identity and E < 0.001 in NCBI-BLAST) as well as in several OTUs in the family Ruminococcaceae and genus *Bacteroides*. At the species level, *Bacteroides ovatus* (37.6 fold, *q* = 0.087), *Ruminococcus lactaris* (2866.7 fold, *q* < 0.001), *Eubacterium oxidoreducens* (3.3 × 10^5^ fold, *q* < 0.001), *Bacteroides xylanisolvens* (47.8 fold, *q* = 0.037), and *Bacteroides acidifaciens* (92.4 fold, *q* = 0.038) were enriched after RS4 intervention.

### RS4 consumption altered faecal SCFAs linked to specific gut microbes

**Figure 4:**
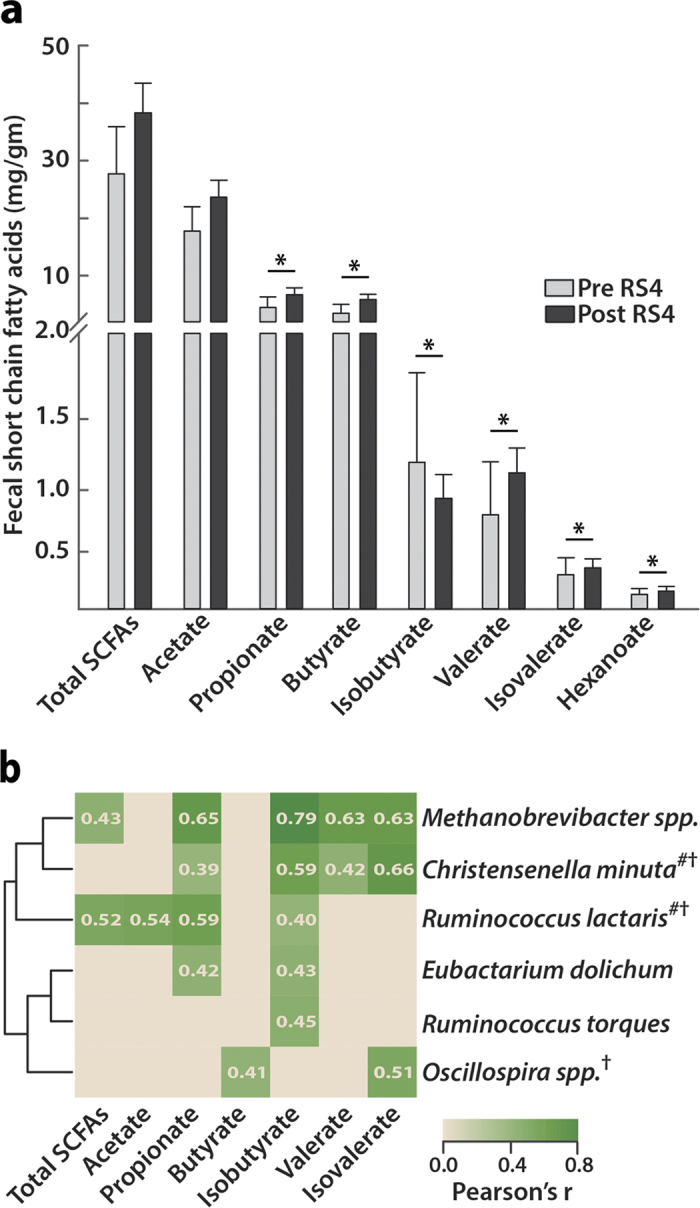
Effects of RS4 on faecal SCFAs. **(a)** SCFA abundance before and after RS4 intervention (*p ≤ 0.05, n = 19). **(b)** Positive correlation of six bacterial species with increased SCFA levels in an RS4-specific manner (all, *p* < 0.05). Pearson coefficients are shown on heat map. #, the closest hit from the NCBI 16S rRNA database cross referenced with the OTU from the Greengenes database. †, species either significantly enriched or approached significance in the RS4 group.

Acetate was the most abundant SCFA, accounting for over 60% of total SCFAs before and after the interventions in both RS4 and CF groups. The individual proportions of the SCFAs, butyric (69.5%, *p* = 0.03), propionic (50.2%), valeric (44.1%), isovaleric (20.3%), and hexanoic (19.2%) acids increased post intervention from baseline in the RS4 group (*p* < 0.05, Fig. 4a and Supplementary Fig. S2) but not in the CF group (data not shown). A 24.6% decrease in isobutyric acid in the RS4 group was observed. A Pearson correlation analysis showed a potential link between significant changes in the gut microbiota composition induced by RS4 and altered SCFA levels (Fig. 4b). Acetate and butyrate levels were correlated (*p* < 0.05) with *Ruminococcus lactaris* (r = 0.54) and *Oscillospira* spp. (r = 0.41). Total SCFAs were correlated with the abundance of two species: *Methanobrevibacter* spp. (r = 0.43) and *Ruminococcus lactaris* (r = 0.52). Propionate and isobutyrate levels were linked to *Methanobrevibacter* spp. (r = 0.65 and r = 0.79, respectively)*, Eubacterium dolichum* (r = 0.42 and r = 0.43, respectively), *Christensenella minuta* (r = 0.39 and r = 0.59, respectively), and *Ruminococcus lactaris* (r = 0.59 and r = 0.40, respectively), of which the latter two were increased by RS4 (Figs 3a,c and 4b). Interestingly, these associations of SCFAs with specific gut microbiota were not observed after CF intervention (data not shown). To our knowledge, prior studies with RS4 have not reported significant SCFA changes in human faecal samples.

### Impact of RS4 intervention on circulatory adipocytokines

In obesity, macrophages infiltrate adipose tissue and secrete proinflammatory cytokines such as IL6 and TNFα^26^. Also, adiponectin is released by adipocytes in the blood and has important roles in lipid and glucose metabolism^27^. Reduced adiponectin levels are associated with various aspects of metabolic dysfunction^28^. Compared with baseline, IL6 decreased by 38% (*p* = 0.04), and adiponectin levels increased by 20% (*p* = 0.002) in the RS4 group, while TNFα did not change significantly. Both TNFα and adiponectin concentrations were lower post RS4 diet compared with post CF diet (*p* = 0.08 and *p* = 0.02 respectively, Table 2). To our knowledge, this is the first report showing changes in adipocytokines, which help determine progression to cardiovascular aberrancies^28^,^29^, in response to RS4 intake in humans.

### Impact of RS4 consumption on body composition, lipids, and glucose metabolism

Individuals had lower % body fat (*p* = 0.05) and lower non-high density lipoprotein (non-HDL, *p* = 0.003), HDL (*p* = 0.005), and total cholesterol (TC, *p* < 0.001) post RS4 consumption compared with post CF consumption (Table 1). A trend was observed for lower waist circumference (*p* = 0.06), glycosylated haemoglobin (HbA1C, *p* = 0.08), and fasting blood glucose (*p* = 0.09) following RS4 consumption compared with CF consumption. It is likely that response variation among participants to an RS4 diet contributed to these higher *p*-values. Varying responses to dietary interventions among individuals are frequently reported^30^. Changes in fasting glucose and HbA1C were more pronounced in this cohort (−8.6% and −1%, respectively) compared with the parent cohort (−4.2% and no decrease, respectively). Attenuation of % body fat combined with a smaller waist circumference indicates a potential reduction in central obesity in these individuals. Although significant, these changes were modest, as measures of body composition do not change rapidly in adults and can take several months to years to show a larger change. Waist circumference, TC, HDL, and non-HDL were also reduced in the RS4 group compared with baseline (all, *p* < 0.05). No significant effects of RS4 were observed on blood pressure or triglyceride levels in either group (Table 2). The average lipid and glycaemic profiles were apparently within normal limits, likely due to prescribed medication usage for various metabolic dysfunctions (Table 2).

### Inter-associations between gut bacteria and metabolic functions

**Figure 5:**
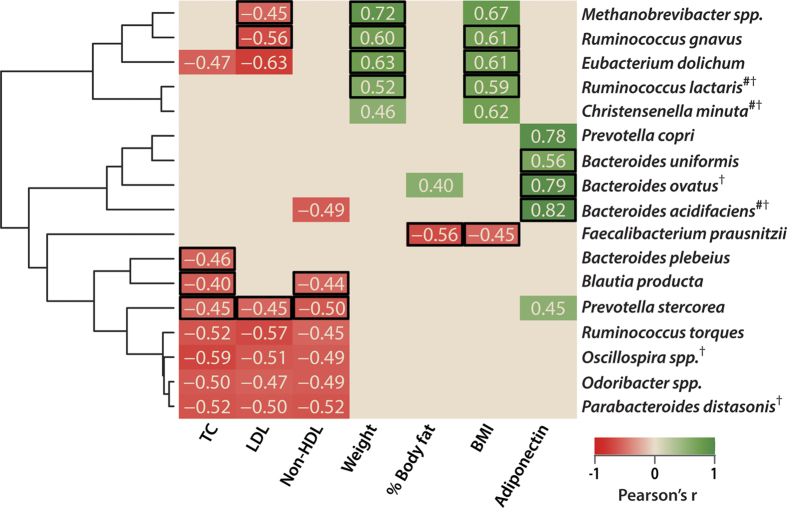
Associations between gut microbiota and host biological parameters after RS4 and CF interventions. **(a)** Heat map showing Pearson’s r values (all, *p* < 0.05). Black rectangular borders indicate an association present only post RS4 intervention. #, the closest hit from the NCBI 16S rRNA database cross referenced with the OTU from the Greengenes database. †, species either significantly enriched or approached significance in the RS4 group, n = 15.

Multiple novel gut microbial associations with metabolic functions were observed post intervention in the RS4 group compared with the CF group (Fig. 5). We propose that the associations detected post RS4 diet, but not post CF diet, could be induced by RS4. However, several associations were common to both groups, lacking specific response to RS4 enrichment. RS4-specific inverse correlations were observed between TC and the abundances of *Bacteroides plebeius* (r = −0.46), *Blautia producta* (r = −0.49), and *Prevotella stercorea* (r = −0.45; all, p < 0.05). Although the abundances of *Parabacteroides distasonis* and *Oscillospira* spp. were enriched post RS4 compared with post CF intervention, their negative association with TC, low density lipoprotein (LDL), and non-HDL were not RS4-specific (all, p < 0.05). In another instance, while RS4 did not significantly alter the abundance of *Faecalibacterium prausnitzii*, an RS4-specific negative correlations between this species and body mass index (BMI, r = −0.45) and % body fat (r = −0.56) were observed (all, p < 0.05). An RS4-specific correlation between adiponectin and *Bacteroides ovatus* (r = 0.79, *p* < 0.01), *Bacteroides uniformis* (r = 0.56, *p* < 0.05), and *Bacteroides acidifaciens* (r = 0.82, *p* < 0.001) was observed (Fig. 5). RS4 intake did not significantly enrich *Methanobrevibacter* spp. and *Eubacterium dolichum*, but these bacteria were correlated with weight and BMI (Fig. 5) as well as with SCFA levels (Fig. 3b) in an RS4-specific manner. *Methanobrevibacter* spp. (r = −0.45), *Ruminococcus gnavus* (r = −0.56), and *Prevotella stercorea* (r = −0.45) were negatively correlated with LDL (*p* < 0.05), while *Blautia producta* (r = −0.44) and *Prevotella stercorea* (r = −0.50) were negatively associated with TC and non-HDL (all, *p* < 0.05) in an RS4-specific way.

### Intra-association within gut microbes

**Figure 6:**
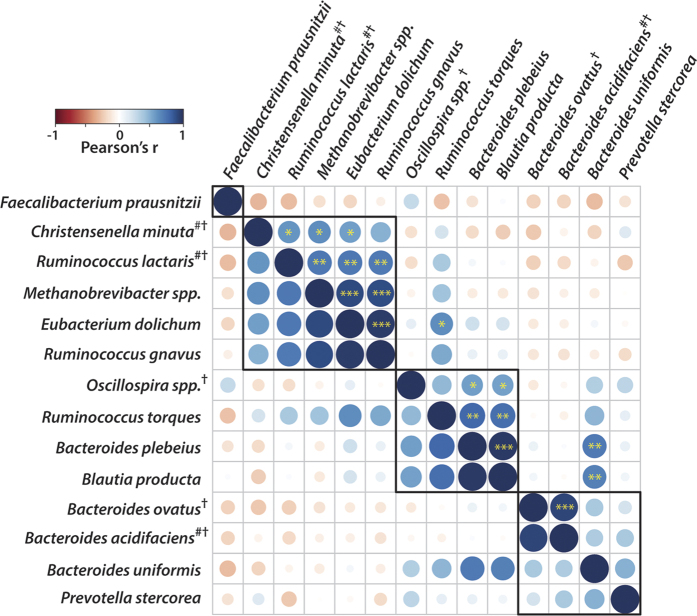
Intra-associations within bacterial species that were correlated with metabolic functions or SCFAs in an RS4-specific manner. Heat map showing Pearson’s r values, corresponding to the size of the circle (n = 19). The black border indicates clustering of species (*p ≤ 0.05, **p ≤ 0.01, ***p ≤ 0.001, shown only in the upper triangle). #, the closest hit from the NCBI 16S rRNA database cross-referenced with the OTU from the Greengenes database. †, species either significantly enriched or approached significance in the RS4 group.

Little is known about how the relative abundance of one microbial species may influence the presence of another species within the gut ecosystem, particularly in response to RS4 consumption. To evaluate this question, we examined intra-association and clustering among those bacteria that showed RS4-specific association with SCFAs and metabolic features. Three *Bacteroides* species that showed a positive correlation with adiponectin and *Prevotella stercorea*, which associated with TC, LDL, and non-HDL, were clustered together (Figs 5 and 6). In general, a higher association within Bacteroidetes species or within Firmicutes species was observed, although there were exceptions. One example is *Bacteroides plebius*, which correlated with the Firmicutes member *Blautia producta* (r = 0.98, *p* = <0.001), and both of these were negatively linked to RS4-induced changes in TC or non-HDL. *Christensenella minuta* tended to associate with *Ruminococcus lactaris* (r = 0.58, *p* = 0.02), and both were enriched after RS4 intervention. Both *Christensenella minuta and Ruminococcus lactaris* also clustered with *Methanobrevibacter spp*. and *Eubacterium dolichum* (both, *p* < 0.05), but not with *Ruminococcus torques* and *Oscillospira spp*., although all of them were associated with one or more SCFAs (Figs 4b and 6).

### Association among metabolic features

Variation in response to RS4 among participants was observed, and the pattern is similar for various host metabolism parameters (response variation data not shown). This pattern may be due to the known link between high levels of circulating lipids and glucose with lower blood adiponectin levels^31^ and the correlation of up-regulated IL6 and tissue necrotic factor-α with the pro-inflammatory state in obesity^32^. The clustering of metabolic dysfunctions and CVD risk factors in adults has been observed in epidemiological studies and in the clinical setting^33^. In line with that, we observed consistent intra-associations among parameters of metabolic dysfunction within our data set independent of dietary changes (Supplementary Fig. S3). TC and non-HDL, but not HDL, correlated more closely with each other (r = 0.95, *p* < 0.001). Similar correlations were observed among various anthropometric measures, such as weight, BMI, and waist circumference, as well as fasting glucose (*p* < 0.05). Fasting glucose correlated with IL6 level (r = 0.51, *p* = 0.07), which in turn was associated with diastolic blood pressure (r = 0.78, *p* < 0.01), systolic blood pressure (r = 0.52, *p* = 0.07), and waist circumference (r = 0.60, *p* = 0.03). Triglyceride concentrations were positively associated with weight (r = 0.82, *p* < 0.001), waist circumference (r = 0.60, *p* = 0.03), and BMI (r = 0.64, *p* = 0.02), while negatively correlated with HDL (r = −0.65, *p* = 0.02) and adiponectin (r = −0.45, *p* = 0.12) but had little apparent link with TC and LDL.

## Discussion

Our present study provides for the first time a microbiome signature in response to RS4 consumption in subjects with MetS. Among the comorbidities of MetS, numerous studies associated a dysbiosis with obesity and type 2 diabetes^34^, but less is known about the role of the microbiota-diet interactions in hypercholesterolemia^35^. A handful of *in vitro*, animal, and healthy volunteer studies suggested that the microbiota affects lipid metabolism^36^,^37^,^38^, but a clear understanding is lacking. Reduction in plasma TC and non-HDL and HDL cholesterol after RS4 consumption was consistently observed in the larger parent cohort^6^ as well as in the present sub-cohort when compared with the control group and with baseline. In addition, a novel link between TC and non-HDL with three bacterial species was observed in an RS4-specific manner. The effect of an RS4 diet on *Parabacteroides distasonis* without any context of lipid metabolism was previously reported^8^ and further confirmed by our results. *Parabacteroides distasonis* was augmented post-RS4 compared with the post-CF and baseline. This species showed a correlation with TC and non-HDL in both intervention groups and clustered with species belonging to both the Bacteroidetes and Firmicutes phyla. While RS4 also lowered HDL, such a reduction is not always associated with increased cardio-vascular disease risk, while lowering of TC and non-HDL, which includes LDL, remains critical^39^.

Another novel RS4-induced microbial enrichment involved *Christensenella minuta* in the family Christensenellaceae, which was identified in human faeces only recently^40^. This species was reported as being heritable, based on host genetics, and abundant in healthy, lower-BMI individuals, and its addition reduced microbiome-mediated weight gain in germ-free mice^41^. *Christensenella minuta* was enriched post RS4-diet compared with post CF-diet and the baseline, and correlated in an RS4-specific manner with higher propionate, isobutyrate, valerate, and isovalerate concentrations, similar to that of *Methanobrevibacter* spp. in the Methanobacteriaceae family. This result is in line with a previous report that this bacterium co-occurs with Methanobacteriaceaea members, and both together produce SCFAs^41^. Moreover, *Christensenella minuta* was reported to augment *Oscillospira*, a bacterium that was enriched and associated with higher isovarelate levels post-RS4 but not post-CF. However, contrary to a prior suggestion^41^, our results present evidence that *Christensenella minuta* is amenable to dietary intervention. We also observed a positive correlation of this species with weight and BMI, which was, however, non-specific to RS4 intervention. Future investigations may address whether *Christensenella minuta*-mediated energy harvest from SCFAs is utilized differently by individuals with MetS than by the lean and healthy individuals involved in prior studies^40^,^41^.

Complex carbohydrate or glycan availability is a major factor in shaping the gut microflora^42^. Since many of the starch-degrading enzymes are represented as a starch-utilization system (SUS) in ~20% of the genome of Bacteroidetes species^42^,^43^,^44^, enrichment of *Bacteroides ovatus* and *Parabacteroides distasonis* after RS4 intervention in this study was as expected. Further confirmation with Carbohydrate-Active Enzymes (CAZy) database (http://www.cazy.org/)^45^ supported that Bacteroidetes species are dominant starch-degrading bacteria after RS4 intervention.

The strengths of this study include statistically significant observations of prebiotic RS4-induced changes across microbial composition, faecal SCFA levels, and host immunometabolic functions, all of which are relevant to the underlying physiology of individuals with pre-existing metabolic dysfunctions. Human dietary interventions conducted within natural settings and reporting statistically significant outcomes that are consistent across a broad range of metabolic health measures are rare^46^. One exploratory human intervention study attempted to show the prebiotic diet concept for combating obesity by undertaking a similar comprehensive investigation, but utilized a parallel design and a different kind of non-RS prebiotic diet. In that study, the changes in the microbiota and some metabolites, although not including SCFA, were observed, but without any concomitant improvement in metabolic functions^47^. This difference could be due to RS4 having a different prebiotic impact on such parameters or due to the crossover design of our study, which allowed each participant to serve as his/her own control, minimizing the influence of confounding variables on treatment effects. In addition, a stringent FDR at 0.05 helped minimize false positives for gut microbial changes. Furthermore, novel associations among host metabolic functions and species-level composition of the gut microbiota were observed, many of which also coincided with intra-species clustering, indicating the possibility of a synergistic (within bacterial species) as well as super-system interactions (symbiotic host–microbe systems) linked with metabolic functions. However, we note that faecal SCFAs represent <5% of the total SCFAs that are typically excreted, while the major portion of SCFAs is efficiently absorbed in the intestinal lumen^36^. Also microbial communities in faecal samples may potentially exclude gut residents that are not shed. Nevertheless, faecal specimens are the most practical samples obtained from a human dietary intervention study for assessing the gut microbiome.

In conclusion, this study provides evidence that dietary RS4 supplementation selectively changes the gut microbial and metabolite environment as well as associated host metabolic functions. To our knowledge, this is the first holistic study that investigated the effects of the fermentable fibre, RS4, on the gut microbial ecology, functional metabolites like SCFAs, and physiological responses in the host in one well-designed study in a free living Caucasian cohort with signs of MetS. The findings support the perceived role of the microbiota–host interaction in nutritional therapies with important implications for dietary guidelines for individuals with metabolic disorders, a major public health concern of the present day.

## Additional Information

**How to cite this article**: Upadhyaya, B. *et al*. Impact of dietary resistant starch type 4 on human gut microbiota and immunometabolic functions. *Sci. Rep*. **6**, 28797; doi: 10.1038/srep28797 (2016).

## Supplementary Material

Supplementary Information

Supplementary table S2

## Figures and Tables

**Table 1:** Estimated nutrients intake at baseline and at the end of intervention periods^a^.

Nutrients	BL	Post CF	Post RS4	*p*: post CF vs post RS4
Caloric intake (kcal/d)	1774 ± 154	1528 ± 121	1716 ± 128	NS
Protein (g/d)	76 ± 7	72 ± 5	62 ± 4	NS
Carbohydrate (g/d)	218 ± 21	220 ± 19	212 ± 18	NS
Total fat (g/d)	68 ± 7	60 ± 5	53 ± 5	NS
Cholesterol (mg/d)	415 ± 40	442 ± 38	407 ± 35	NS
Saturated fat (g/d)	25 ± 3	22 ± 2	21 ± 2	NS
Monounsaturated fat (g/d)	26 ± 3	22 ± 2	19 ± 2	NS
Polyunsaturated fat (g/d)	10 ± 1	9 ± 1	7 ± 1	NS
Total dietary fibre (g/d)	18 ± 2	16 ± 2	27 ± 2	**<0.001**

^a^Data are Least Square Means ± SEM adjusting for age, sex, season, colony and baseline value analysed by linear mixed model; *n* = 18–20 due to missing data points.

BL: baseline; CF: control flour; RS4: resistant starch type 4.

**Table 2:** Means of biological parameters at baseline and at the end of intervention periods^a^.

	BL	Post CF	Post RS4	*p:*Post CF vs Post RS4	*p:*BL vs post RS4
***Anthropometrics***					
Weight (kg)	90.9 ± 3.4	91.0 ± 0.4	91.6 ± 0.4	NS	NS
BMI (kg/m^2^)	32.8 ± 1.1	32.8 ± 0.1	32.7 ± 0.1	NS	NS
Waist (cm)	109.0 ± 2.8	108.8 ± 0.9	106.6 ± 0.9	**0.06**	**0.02**
Systolic BP (mm Hg)	135.0 ± 3.9	134.6 ± 3.5	137.5 ± 3.5	NS	NS
Diastolic BP (mm Hg)	73.7 ± 2.2	68.6 ± 2.0	73.3 ± 2.0	NS	NS
% Body Fat	37.0 ± 1.8	37.7 ± 0.3	37.3 ± 0.3	**0.05**	NS
Fat-free mass (kg)	58.8 ± 3.0	58.8 ± 0.3	58.9 ± 0.3	NS	NS
***Glycemic Variables***(***mg/dL***)					
Fasting glucose	106.5 ± 4.1	111.5 ± 4.2	101.9 ± 4.3	**0.09**	NS
Postprandial glucose	113.5 ± 11.8	124.3 ± 7.3	114.3 ± 7.5	NS	NS
HbA1C (% of total Hb)	5.89 ± 0.3	5.81 ± 0.1	5.75 ± 0.1	**0.08**	NS
***Lipid Variables***(***mg/dL***)					
Total cholesterol	196.6 ± 11.6	192.8 ± 4.6	187.8 ± 0.9	**<0.001**	**0.01**
HDL cholesterol	43.6 ± 3.3	44.1 ± 1.3	39.8 ± 1.3	**<0.01**	**0.001**
LDL cholesterol	122.7 ± 10.1	117.4 ± 5.6	118.0 ± 6.1	**0.06**	**0.06**
NonHDL cholesterol	153.1 ± 11.8	148.4 ± 4.6	147.5 ± 4.9	**<0.01**	**0.03**
TC/HDL (ratio)	5.0 ± 0.5	4.7 ± 0.2	5.1 ± 0.2	NS	NS
Triglycerides^b^	161.5 ± 19.9	144 (119–176)	138 (110–173)	NS	NS
***Blood Biomarkers***					
IL6 (pg/mL)	1.3 ± 0.2	1.0 ± 0.2	0.8 ± 0.2	NS	**0.04**
TNF-α (pg/mL)	7.9 ± 4.2	9.9 ± 1.2	6.0 ± 1.3	**0.08**	NS
Adiponectin (μg/mL)	8.3 ± 1.5	10.8 ± 0.4	10.0 ± 0.4	**0.02**	**<0.01**

^a^Data are Least Square Means ± SEM adjusting for age, sex, season, colony and baseline values.

^b^Geometric mean and confidence interval are given for log-transformed triglyceride endpoints.

*p*-value ≤ 0.05 were considered significant, between 0.05 and 0.09 considered as approaching significant (trend), when greater than 0.09 is shown as NS (non-significant); Linear mixed model analysis was used to determine significance between post-CF and post-RS4, paired t-test for baseline vs post-RS4, *n* = 18 to 20 due to missing data points. BL: baseline; CF: control flour; RS4: resistant starch type 4; BMI: body mass index; BP: blood pressure; Hb: haemoglobin; HDL: high density lipoprotein; LDL: low density lipoprotein; TC: total cholesterol; IL6: interleukin 6; TNF-α: tissue necrotic factor alpha.
