# Supplementary material for: Impact of dietary resistant starch type 4 on human gut microbiota and immunometabolic functions
Source: Sci Rep. 2016 Jun 30;6:28797. doi: 10.1038/srep28797 (PMC4928084; doi:10.1038/srep28797)

**SUPPLEMENTARY INFORMATION**

# Impact of dietary resistant starch type 4 on human gut microbiota and immunometabolic functions

Bijaya Upadhyaya[1], Lacey McCormack[1#], Ali Reza Fardin-Kia[2#], Robert Juenemann[1#], Sailendra Nichenametla[1], Jeffrey Clapper[3], Bonny Specker[4], and Moul Dey[1*]

[1]Health and Nutritional Sciences, South Dakota State University, Box 2203, Brookings, SD 57007, USA

[2]Office of Regulatory Science, Center for Food Safety and Applied Nutrition, HFS-717, US Food and Drug Administration, College Park, MD 20740, USA

[3]Department of Animal Science, South Dakota State University, Box 2170, Brookings, SD 57007, USA

[4]Ethel Austin Martin Program in Human Nutrition, South Dakota State University, Box 506, Brookings, SD 57007, USA

[#] Equal contributors

* Corresponding author

This file contains additional methods, figures and tables.

## Materials and Methods (Supplementary Information)

Subjects

Participants in this study included a subset of the parent cohort consisting of 20 individuals who originally participated in a dietary intervention with RS4 [1]. Briefly, this study was conducted in an adult population, consisting of both males and females, from two Hutterite colonies in eastern South Dakota, USA. The Hutterites are a culturally homogeneous Caucasian population of Central European ancestry. The parent trial is registered in clinicaltrials.gov (NCT01887964). From 40 participants who had signs of metabolic syndrome at baseline, 26 subjects submitted stool samples at all four data collection time points. Out of 26, 20 subjects (10 from each of the two colonies) were included in the current investigation as their faecal samples were adequate to carry out both sequencing and short chain fatty acid (SCFA) analyses (Fig. 1). The selected cohort included 12 females and eight males, aged 32-77 (Supplementary Table S1). Exclusion criteria included pregnancy, lactation, long-term antibiotic therapy, immune compromised state, cancer, and other conditions that would affect the ability to provide informed consent or comply with the protocol.

Test Diets

Participants consumed control flour (CF) and resistant starch type 4(RS4) flours as described in Nichenametla *et al.* [1]. RS4 flour was made by substituting 30% (v/v) of the CF with RS4 (Fibersym RW, MGP Ingredients Inc., Atchison, KS). The sequence of flour consumption was randomly assigned to the colonies. Dietary intake was assessed by using a 3-day (2 weekdays and 1 weekend day) semi-quantitative food-frequency questionnaire, customized to include foods that Hutterites frequently consume, mostly consisting of items cooked from raw

ingredients. Evidence show that the Hutterites consume more fresh produce in summer and preserved or frozen food in winter resulting into a diet-influenced shifts in microbiome [2]. Since the study period did not overlap with winter months, the impact of seasonal variation on gut microbiome was not considered. Diet analysis was performed using Nutritionist Pro (Axxya Systems, Redmond, WA, USA) to obtain macro-nutrient breakdown of the consumed diets.

Study design:

The original trial [1] was conducted as a two-period, total 26-week long double blind (participants–investigators), placebo-controlled, cluster cross-over intervention (Fig. 1a). Each intervention period (CF and RS4) was for 12 weeks with a washout time of two weeks in between. All data and bio specimens were collected on-site from both colonies at baseline, 12, 14, and 26 weeks except body composition analyses, which were carried out at baseline, 12 and 26 weeks. A modified CONSORT flow chart is provided (Fig. 1b). The study was conducted under free-living conditions, and no dietary restrictions were imposed. Gastrointestinal symptoms and stool consistency were scored based on a one-on-one interview with the participants [1]. Current and past medical information were obtained at each visit which included medication or dietary supplement use, menopausal status, medical conditions, and hospital stays. Seven-day physical activity-recall questionnaires were administered by trained staff to assess any atypical physical activity.

Anthropometric measurements

Height, weight, waist circumference, and blood pressure were measured at the beginning and at the end of each intervention period following standard procedures that were previously described

[3]. Briefly, height was determined using a stadiometer and recorded to the nearest 0.5 cm. Body weight was measured on an electronic scale (Seca GmbH & Co., Hamburg, Germany) with the subjects in light clothes and no shoes and was recorded to the nearest 0.1 kg. Waist circumference was measured at the navel using a tension-sensitive, non-stretching Gulick tape and recorded to the nearest 0.5 cm. Blood pressure was measured with a digital sphygmomanometer in a sitting position. Body composition (fat mass and fat-free mass) was determined by total-body, dual-energy, x-ray absorptiometry (DXA) scanning (Hologic QDR Discovery A, Waltham, MA) as described previously [4]. Fat mass is presented as % body fat in paper.

Blood biochemistry

Overnight fasting blood was collected by venepuncture in vacutainer tubes (BD Biosciences, Franklin Lakes, NJ). Fasting blood glucose, total cholesterol (TC), Low-Density-Lipoprotein cholesterol (LDL), High-density-Lipoprotein cholesterol (HDL), non-HDL cholesterol (non-HDL), triglycerides were determined using the Cholestech LDX point-of-care analyzer (Alere Inc, Waltham, MA), lipid profile and glucose cassettes (Lipid Profile GLU, Alere Inc, Waltham, MA), following the manufacturers' instructions. Postprandial glucose was determined 2 h after either breakfast or the noon meal by using a FreeStyle Freedom Lite blood glucose meter (Abbott Diabetes Care Inc, Alameda, CA), following the manufacturer's instructions. Participants typically consumed a food item made with test flour during the meal prior to the postprandial glucose test. Glycosylated haemoglobin (HbA1C) was determined by an enzyme-based colorimetric assay using the Human HbA1C kit (Crystal Chem, Downers Grove, IL), following the manufacturer's instructions. Each sample was run in duplicate, and a batch control

was used to account for inter-assay variation. HbA1C levels were expressed as the percentage of total haemoglobin. Two markers of inflammation, interleukin 6 (IL6) and tissue necrotic factor-α were determined (in duplicate) in serum using Human ELISA Ready-SET-Go kits, following the manufacturer's instructions (eBioscience, San Diego, CA). Plasma adiponectin levels, an indicator of metabolic functions, were detected by Human Adiponectin Radioimmunoassay following the manufacturer's instructions (Linco Research, St. Charles, MO). Inter- and Intra-assay coefficients of variance were 5.0% and 11.7%, respectively.

Faecal SCFA analysis by gas chromatography-mass spectrometry (GC-MS)

To prepare faecal samples for SCFA analyses, 800-1000 mg faecal sample was homogenized and added to a glass tube containing 1 ml of internal standard (2-ethylbutyric acid in 1-butanol, 1 mg/mL). Organic solvent hexane and derivatizing agents, hydrogen chloride 1-butanol or Boron trifluoride 1-butanol (both from Sigma-Aldrich, St. Louis, MO), were then added with vortexing between each addition. Boron trifluoride 1-butanol and hydrogen chloride 1-butanol were used to create butyl-esters for SCFA detection. Samples were then sonicated and placed in water bath (90-100 $^{0}$C) for 1h. Once cooled to room temperature, water and additional hexane were added to the samples and centrifuged at 3000g for two min. The organic layer (~2 mL) was transferred into a sampling vial with 150 µL inserts, finally adding a pinch (~10 mg) of anhydrous sodium sulphate to remove the residual water. Faecal extracts were stored at -20 $^{0}$C until further analysis.

GC-MS analyses were performed with an GC-MS 5977A (Agilent Technologies, Wilmington, DE, USA) and an HP-5MS UI capillary column (30 m x 0.25mm, 0.2 µm thickness, Agilent, Wilmington, DE, USA). Hydrogen was used as carrier gas at 1.9 mL/min constant flow and a

typical injection volume was 1 μL in the split mode (1:10). The separation of SCFA butyl esters was achieved using an oven temperature program as follow: initial elution temperature was 55 $^{0}$C for 4 min then 5 $^{0}$C/min to 120 $^{0}$C and then 20 $^{0}$C/min to 220 $^{0}$C for 10 min. The selective mass detector was operated in the 'single ion monitoring and scan' (SIM/Scan) mode. The source was maintained at 150 $^{0}$C and the electron energy was 70 eV.

<u>Community structure analysis of gut microbes</u>

Stool DNA was extracted using the QIAamp DNA Stool Mini Kit (QIAGEN, Valencia, CA) according to the manufacturer's instructions. All samples were quantified via the Qubit® Quant-iT dsDNA Broad-Range Kit (Invitrogen, Life Technologies, Grand Island, NY). Sample DNAs were sent to Second Genome (South San Francisco, CA) for metagenome sequencing, and operational taxonomic unit (OTU) identification. Briefly, samples were enriched for bacterial 16S V4 rDNA region, DNA was amplified utilizing fusion primer designed against the Illumina (San Diego, CA) flow cell adapters and indexing barcodes. Each sample was PCR-amplified with two differently bar coded V4 fusion primers. For each sample, amplified products were concentrated using a solid-phase reversible immobilization method for the purification of PCR products and quantified by electrophoresis using an Agilent 2100 Bioanalyzer® (Agilent Technologies, Santa Clara, CA). Sequencing was carried out using Illumina MiSeq platform following standard protocols for 250 cycles with custom primers designed for paired-end sequencing. Using Quantitative Insights Into Microbial Ecology or QIIME [5] and generated custom scripts (Second Genome), sequences were quality-checked and demultiplexed to determine exact matches to the supplied DNA barcodes. Resulting sequences were then searched against the Greengenes reference database of 16S rRNA gene sequences, clustered at 97% by

uclust (closed reference OTU picking). The longest sequence from each OTU was then assigned taxonomic classification via Mothur's Bayesian classifier, trained against the Greengenes database clustered at 98%. For unidentified Greengenes OTUs, we cross referenced with closest hits from NCBI 16S rRNA database with query cover (>90%), identity (>87%), and E value (<0.01).

Statistical analyses

Data analyses were performed using two approaches. Effects of CF and RS4 interventions were determined by comparing end-point data, while effects of RS4 intervention alone was presented by comparing the data before and after RS4 intervention. Linear mixed effects models (SAS MIXED procedure) were used to compare the effects of RS4 and CF on biological parameters. All models included variables for colony and season, where colony was a surrogate for randomization sequence and season was a surrogate for crossover treatment period. Because the crossover design used only two clusters (colonies), and any carryover effect might be confounded by cluster sampling effect (colony effects), thus a carryover effect could not be independently evaluated in the mixed models. Covariates for age, sex, baseline value of the outcome variable, change in total calorie intake were evaluated for their association with the response variables, and covariates that significantly affected at least one outcome variable were retained in the final model as fixed effects. The final models included season, colony, sex, age, and initial baseline value of the response variable as fixed effects.

When appropriate data were presented for either all-participants or stratifying participants based on missing data or outliers in microbial changes as our observations concur with a prior report of

large inter-subject variations in response to RS4 [6]. For microbiome analyses one subject was excluded (n=19) because of *Lactobacillus* probiotic supplement use during the RS4 intervention period. For multivariate analysis PhyCA-Stats™ software package (Second Genome Inc, South San Francisco, CA) was utilized. Differences in the bacterial taxa and host phenotypes among time points were determined by paired t-test or student's t-test as appropriate. If the data were not normally distributed, values were subjected to logarithmic transformations as indicated.

Two approaches for sampling normalization were employed to account for uneven sequencing depth during data analyses on metagenome sequencing. First, taxa were filtered to those present in at least one of the samples, selecting 55,079 sequences from each sample before calculating community-wide dissimilarity measures. This facilitated calculations of microbial relative abundances (Fig. 3b, 4b, 5, 6, and Supplementary Fig. S1), and principal coordinate analyses (Fig. 2). Second, the samples were normalized to 1 million counts. Thus the shift in microbial profile between the samples or groups was calculated based on per million sequences, which facilitated calculations of 71 differentially abundant OTUs (Supplementary Table S2) or species (Fig. 3a and 3c).

We used various R-packages (http://www.r-project.org/) for downstream data analyses on gut microbiome. Package edgeR (http://www.bioconductor.org/packages/release/bioc/html/edgeR.html) [7] which has the distinct benefit of increasing the sensitivity of detecting differentially abundant features, was utilized to control for inter-subject variability because of a model-based normalization to adjust for varying sequencing depths. This approach of identifying differentially abundant taxa between treatments

borrows from the RNA sequencing literature testing for differential gene expression. These methods have recently been demonstrated to effectively translate to microbiome analyses [8], offering a robust method with options for paired sample comparisons even for experiments with minimal levels of biological replication. To correct for multiple comparisons, a false discovery rate correction was used to correct for *p*-values (adjusted *p* is represented as *q*). In order to generate correlation matrices and heat maps, several R-packages, namely Hmisc (http://CRAN.R-project.org/package=Hmisc) [9], corrplot (http://CRAN.R-project.org/package=corrplot) [10], gplots (http://CRAN.R-project.org/package=gplots) [11], and ecodist (https://CRAN.R-project.org/package=ecodist) [12] were used. Significant variation in microbiome baseline values between intervention-groups were ruled out using a Permutational Multivariate Analysis of Variance that also confirmed the effectiveness of the washout period. Two-dimensional Principal Coordinate Analysis (PCoA) followed by Adonis test was used for plotting dissimilarity values so that the similar data points get closer to each other. Statistical analyses were carried out using Sigma Plot software (Systat Software, Inc., San Jose, CA) and the data were presented as means±S.E.M, unless otherwise noted. A *p* value of 0.05 or less was considered significant, while a *p* value of 0.05 to 0.09 was considered trend or approaching significance.

## References Cited in the Supplementary Information


1 Nichenametla, S. N. *et al.* Resistant starch type 4-enriched diet lowered blood cholesterols and improved body composition in a double blind controlled cross-over intervention. *Mol Nutr Food Res* **58**, 1365-1369, doi:10.1002/mnfr.201300829 (2014).

2 Davenport, E. R. *et al.* Seasonal variation in human gut microbiome composition. *PLoS One* **9**, e90731, doi:10.1371/journal.pone.0090731 (2014).

3 Wey, H. E., Binkley, T. L., Beare, T. M., Wey, C. L. & Specker, B. L. Cross-sectional versus longitudinal associations of lean and fat mass with pqct bone outcomes in children. *J Clin Endocrinol Metab* **96**, 106-114, doi:10.1210/jc.2010-0889 (2011).

4 Wosje, K. S., Binkley, T. L., Fahrenwald, N. L. & Specker, B. L. High bone mass in a female hutterite population. *J Bone Miner Res* **15**, 1429-1436, doi:10.1359/jbmr.2000.15.8.1429 (2000).

5 Caporaso, J. G. *et al.* Qiime allows analysis of high-throughput community sequencing data. *Nat Methods* **7**, 335-336, doi:10.1038/nmeth.f.303 (2010).

6 Martinez, I., Kim, J., Duffy, P. R., Schlegel, V. L. & Walter, J. Resistant starches types 2 and 4 have differential effects on the composition of the fecal microbiota in human subjects. *PLoS One* **5**, e15046, doi:10.1371/journal.pone.0015046 (2010).

7 Robinson, M. D., McCarthy, D. J. & Smyth, G. K. Edger: A bioconductor package for differential expression analysis of digital gene expression data. *Bioinformatics* **26**, 139-140, doi:10.1093/bioinformatics/btp616 (2010).

8 McMurdie, P. J. & Holmes, S. Waste not, want not: Why rarefying microbiome data is inadmissible. *PLoS Comput Biol* **10**, e1003531, doi:10.1371/journal.pcbi.1003531 (2014).

9 Harrell Jr, F. E. & Dupont, C. Hmisc: Harrell miscellaneous. R package version 3.17-0. (2015).

10 Wei, T. Corrplot: Visualization of a correlation matrix. R package version 0.73. (2013).

11 Warnes, G. R. *et al.* Gplots: Various r programming tools for plotting data. R package version 2.17.0. (2015).

12 Goslee, S. C. & Urban, D. L. The ecodist package for dissimilarity-based analysis of ecological data. *J Stat Softw* **22**, 1-19 (2007).

**Supplementary Table S1. Baseline characteristics of 20 participants**

| Criteria | *n* |
|---|---|
| ***Age (years)*** | |
| <50 | 5 |
| ≥50 | 15 |
| ***BMI*** | |
| <30 | 5 |
| ≥30 | 15 |
| ***Gender*** | |
| Male | 8 |
| Female | 12 |
| ***Medication for*** | |
| No medication | 8 |
| Type 2 diabetes | 4 |
| Heart diseases | 2 |
| Blood pressure | 11 |
| Fibre supplement | 2 |
| Probiotic supplement | 1 |
| Digestive support | 3 |
| Cholesterol lowering | 5 |

*n:* number of individuals

BMI: body mass index

**Supplementary Table S2: 71 differentially modulated operational taxonomic units (OTUs) after RS4 intervention compared to CF control (a separate Excel file)**

**<u>Figures and Legends</u>**

**Supplementary Figure S1. Phylum-level gut microbial composition of participants at baseline**. Mean percentage of total bacteria in the major phyla (n=19).

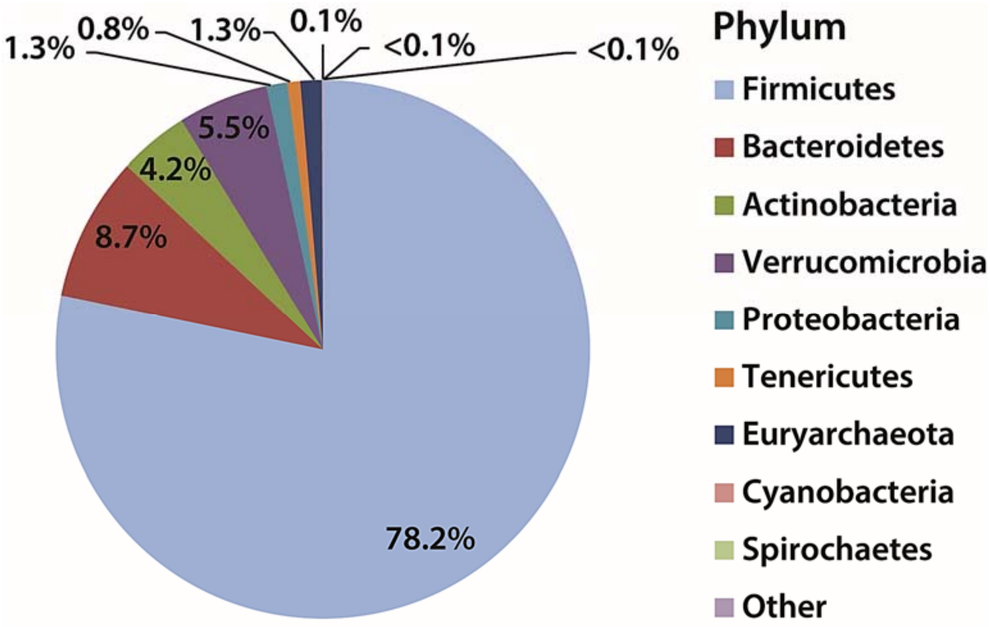

**Supplementary Figure S2: Representative chromatogram showing separation of the butyl esters of SCFA fragment in a baseline sample.** Butyl esters of SCFAs, acetic acid (1), propionic acid (2), iso-butyric acid (3), butyric acid (4), iso-valeric acid (5), valeric acid (6), internal standard (7), and hexanoic acid (8) clearly separated out long chain saturated fatty acid- and mono-unsaturated fatty acid-esters (9). * represents the di-butyl ether as a by product of butylation that did not co-elute with the sample analytes.  Samples were run using Scan/SIM mode; the SIM mode facilitated quantification and the scan mode was used for screening and visualization.

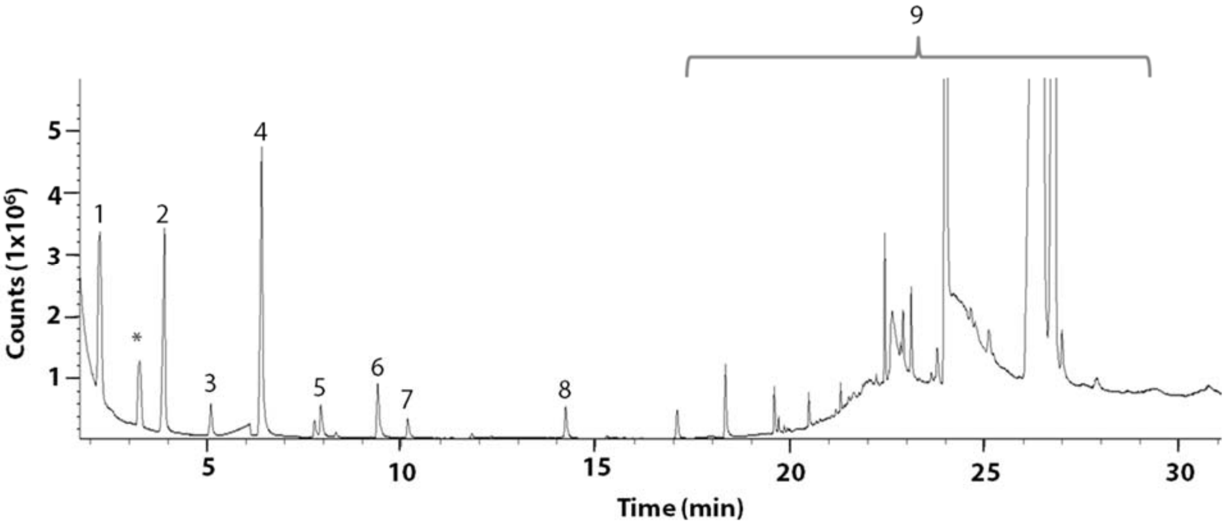

**Supplementary Figure S3. Associations among host biological parameters in the RS4 group**. Heat map showing Pearson's r values, corresponding to the size of the circle (n=13, *$p \le 0.05$, **$p \le 0.01$, ***$p \le 0.001$).

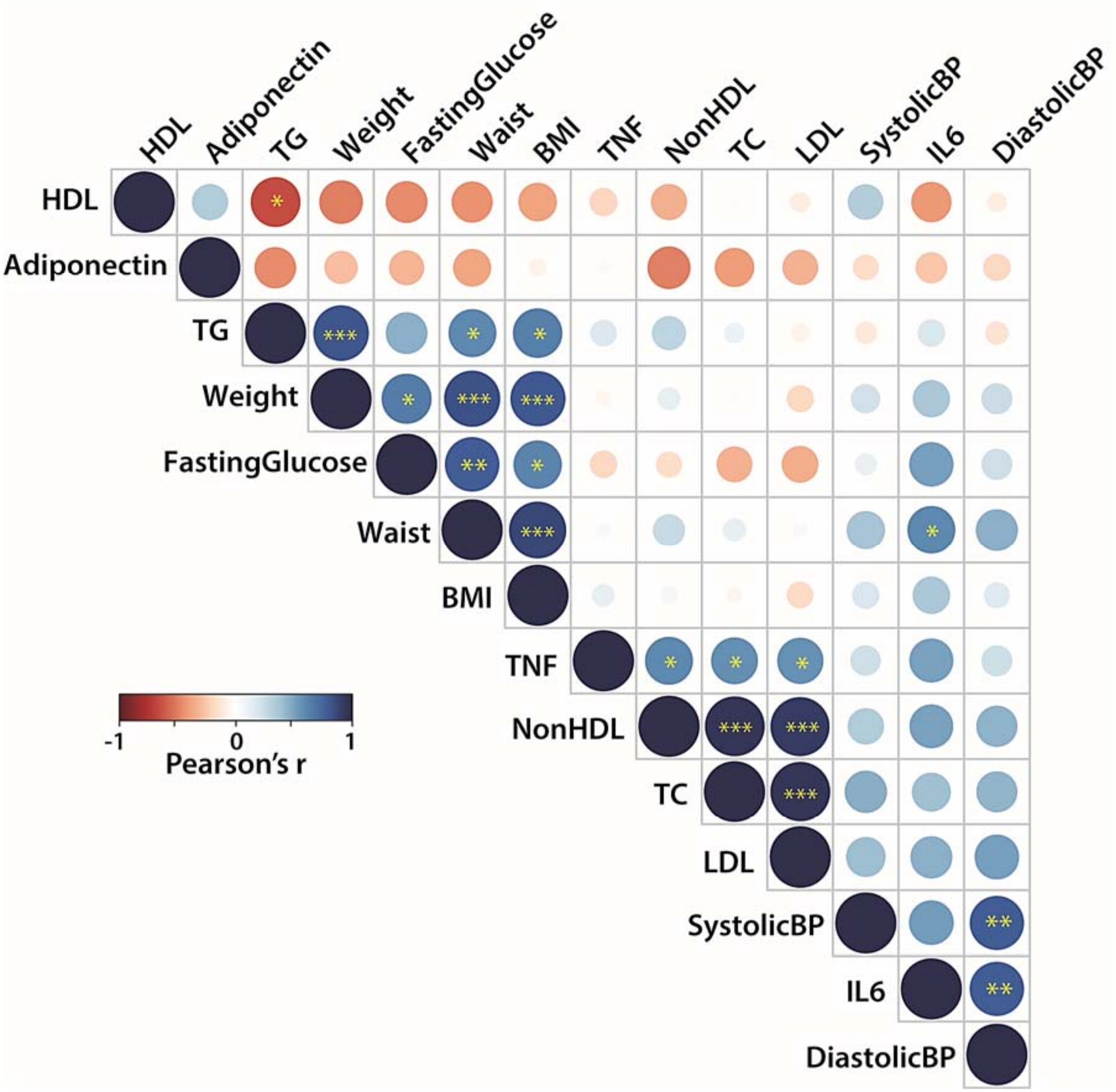

Supplement: Supplementary Information [file srep28797-s1.pdf]
